# Genome-Wide Transcript Profiling Reveals Novel Breast Cancer-Associated Intronic Sense RNAs

**DOI:** 10.1371/journal.pone.0120296

**Published:** 2015-03-23

**Authors:** Sang Woo Kim, Elane Fishilevich, Gustavo Arango-Argoty, Yuefeng Lin, Guodong Liu, Zhihua Li, A. Paula Monaghan, Mark Nichols, Bino John

**Affiliations:** 1 University of Pittsburgh School of Medicine, Pittsburgh, Pennsylvania 15260, United States of America; 2 Department of Neurobiology, University of Pittsburgh, 3501 Fifth Ave, Pittsburgh, Pennsylvania 15260, United States of America; 3 Department of Pharmacology and Chemical Biology, University of Pittsburgh School of Medicine, Pittsburgh, Pennsylvania 15260, United States of America; University of Alabama at Birmingham, UNITED STATES

## Abstract

Non-coding RNAs (ncRNAs) play major roles in development and cancer progression. To identify novel ncRNAs that may identify key pathways in breast cancer development, we performed high-throughput transcript profiling of tumor and normal matched-pair tissue samples. Initial transcriptome profiling using high-density genome-wide tiling arrays revealed changes in over 200 novel candidate genomic regions that map to intronic regions. Sixteen genomic loci were identified that map to the long introns of five key protein-coding genes, CRIM1, EPAS1, ZEB2, RBMS1, and RFX2. Consistent with the known role of the tumor suppressor ZEB2 in the cancer-associated epithelial to mesenchymal transition (EMT), *in situ* hybridization reveals that the intronic regions deriving from ZEB2 as well as those from RFX2 and EPAS1 are down-regulated in cells of epithelial morphology, suggesting that these regions may be important for maintaining normal epithelial cell morphology. Paired-end deep sequencing analysis reveals a large number of distinct genomic clusters with no coding potential within the introns of these genes. These novel transcripts are only transcribed from the coding strand. A comprehensive search for breast cancer associated genes reveals enrichment for transcribed intronic regions from these loci, pointing to an underappreciated role of introns or mechanisms relating to their biology in EMT and breast cancer.

## Introduction

Advances in gene expression profiling using technologies such as whole genome tiling arrays (GTAs) and deep-sequencing (“next-gen”) within the last decade have resulted in the unexpected realization that ~90% of the human genome is transcribed [1, 2]. While many important classes of non-protein coding RNAs (ncRNAs) have been identified, ranging from small RNAs such as microRNAs (miRNAs) to long noncoding RNAs (lncRNAs) [3], to extraordinarily long ncRNAs such as the 108 kb *Air* transcript [4], the functions of most ncRNAs remain unknown [5].

One approach to discover important RNAs is to identify those that are dysregulated in diseases. While genes can associate with a disease as a side-effect of an aberrant mechanism, minimally, the identification of disease-associated RNAs can lead to the discovery of new molecular mechanisms that can aid in the diagnosis or prognosis of the disease, and sometimes lead to a therapeutic path [6]. For example, transcriptome sequencing in cancer tissues has led to the discovery of novel ncRNAs such as PCAT-1, a specific regulator of cell proliferation [7] and chimeric, cancer-associated RNAs [8].

Breast cancer treatment has been revolutionized in the recent decades with the use of individual biomarkers such as ERα and HER2/neu [9]. Gene expression profiling studies in the past decade have also led to the development of multi-gene biomarkers [9] such as MammaPrint, a 70 gene diagnostic test used to evaluate whether patients would benefit from chemotherapy [10]. In addition to the clinically established biomarkers that are all based on protein-coding genes, several new promising candidate biomarkers have been found to correlate with breast cancer. Dozens of lncRNAs from the HOX gene cluster were recently found to have reduced expression in breast cancer [11], while other HOX lncRNAs were detected in primary tumors but not in metastatic breast cancer. The metastasis-associated HOTAIR, a 2.2 kb long intergenic ncRNA, is overexpressed in metastases, and its high level of expression in primary breast tumors is a predictor of metastasis and death [11]. These results may lead to the identification of new biomarkers, better understanding of molecular classification of breast cancer sub-types [12].

To identify biomarkers for breast cancer, we conducted an unbiased screen of the human transcriptome. We used GTAs for genome-wide expression profiling to identify genes that were altered in cancer versus control human breast cancer samples. Approximately 200 potential breast-cancer markers were identified and validated by quantitative PCR. This study focuses on 16 genomic regions that were significantly (*p*<0.001) misregulated in tumor samples. Changes in expression were validated by RNA in situ hybridizations (ISH) to compare the localization patterns in normal and tumor breast tissues. ISH experiments not only confirm the observed down-regulation of these RNAs in tumor, but also reveal that some of these transcripts, such as those deriving from ZEB2 introns, are down-regulated in epithelial tumor cells. ZEB2 is a tumor suppressor gene and is also required during embryogenesis, particularly for neural crest formation [13]. We examined the expression of ZEB2 intronic regions in developing mice. Intronic ZEB2 RNAs are not only conserved sequences in mice, but also have strong tissue-specific expression in the mouse brain, with strongest expression in the cortical plate, a pattern that is similar yet distinct from the canonical ZEB transcript. These data suggest that these regions may be alternatively-spliced transcripts or novel transcripts from within known genomic regions. To differentiate these possibilities, we performed paired end sequencing and found that the expressed intronic regions do not have protein coding potential and are frequently transcribed in a pattern that resembles distinct, yet defined transcriptional units, supporting the idea that the identified loci correspond to novel ncRNAs and variants. Our results identify novel RNAs from intronic regions that associate with breast cancer and embed a highly complex, transcriptional landscape that is misregulated in cancer.

## Materials and Methods

### Animals and cell culture

Timed pregnant mice were purchased from Charles River Laboratories. Mothers were euthanized by CO_2_ inhalation and embryos were collected via cesarean section at E14 and E16. Brains from embryos were dissected prior to fixation. Embryos were fixed in 4% paraformaldehyde (PFA; pH7.4). Animal protocols and procedures were approved by the Institutional Animal Care and Use Committee at the University of Pittsburgh (protocol numbers: #1102130, #1001013A) and adhered to the National Institutes of Health guidelines. For cell culture, MCF7 and MDA-MB 231 breast adenocarcinoma cell lines were grown at 37°C with 5% CO_2_ in RPMI and DMEM (GIBCO), respectively, with 10% FBS and 0.5% penicillin/streptomycin.

### Genome-wide tiling array analysis

Matched pair, total RNA samples for genome tiling array (GTA) analyses were purchased from Ambion (patients 1–2, Samples: 01070141 & 01070151) and Asterand plc (patients 3–4, Samples: 17082A1/B1 & 19379A1/B1). To profile genome-wide expression patterns in those four patients, eight independent high-density GeneChip Human Tiling 1.0R Array sets (Affymetrix) were used to interrogate the expression of ~40 million distinct genomic locations that cover non-repetitive regions of the human genome. Each high-density microarray set consisted of 14 slides with 25-nt long probes that target the majority of the human genome, tiled at an average resolution of 35 nts. To minimize operator biases, cDNA library preparation from total RNA and downstream microarray hybridization as recommended by the manufacturer were performed at two independent facilities, Asuragen (Austin, TX) and GPCL (University of Pittsburgh) using whole Transcript Double-Stranded Target Assay for patients 1–2, and patients 3–4. The resulting data were analyzed using MAT [14] to detect candidate genomic regions that are differentially expressed. The number of candidate hits was reduced by focusing on regions that manifest either high evolutionary conservation as reported by UCSC genome browser or detectable similarity (BLAST e-value <0.001) to mRNAs known to be involved in cancer.

### Real-Time PCR

Primers (S1 Table) for amplification were designed using Real-Time PCR Assay Design Tool from Integrated DNA Technologies. Total RNA (1μg) was reverse-transcribed with random primers using the iScript Select cDNA Synthesis Kit (Bio-Rad). Quantitative PCR (qPCR) amplification was performed using Maxima SYBR Green qPCR Master Mix with ROX Reference Dye (Fermentas), in 96-well plates (ABI) using Stratagene MX3000P Real-Time PCR System. The PCR conditions were as follows: 10 min at 95°C, followed by 40 cycles of: 95°C for 30 sec, 60°C for 1 min, and 72°C for 1 min. The data were normalized to three housekeeping genes (GAPDH, ACTB, and RPLP0) and relative changes in gene expression were quantified using the ΔΔC_T_ method [15].

### Riboprobe construction

Riboprobes were transcribed with either T7 or SP6 RNA polymerase using DIG RNA labeling kit (Roche or Promega) per manufacturer instructions. Unincorporated nucleotides were removed using Illustra ProbeQuant G-50 Micro Columns (GE Healthcare). The riboprobes were diluted (1:1) with hybridization buffer, stored at -20°C. 1–2μl of each riboprobe was used per slide for RNA ISH. Probes used in riboprobe-based assays as well as experiments involving oligonucleotide probes that bind to a target RNA were designed using non-repetitive sequences, minimizing overlap with other regions of the target genome using NCBI BLAST.

### RNA *In-situ* Hybridization

Tissues for RNA in situ hybridization and immunohistochemical analyses were obtained from the University of Pittsburgh Health Sciences Tissue Bank. These de-identified samples were collected by surgical resection from patients diagnosed with invasive ductal carcinoma. Mouse tissue was derived from CD1- timed pregnant animals. The day of the vaginal plug was designated E0.5. The human tissue was fixed in 60% ethanol, 30% of 27% formaldehyde, and 10% glacial acetic acid. Mouse tissue was fixed in 4% paraformaldehyde pH7.4. Fresh mouse brains and fresh-frozen human breast tissues were placed directly into the fixative and incubated at 4°C overnight. The tissue was washed three times (30 min each) in 60% ethanol in water at room temperature and left overnight in 70% ethanol at 4°C. The tissue was then dehydrated through an ethanol series 80%, 95% ethanol, 100% ethanol (2), cleared in 1:1 ethanol:Xylenes (30 min), and Xylenes (overnight) and embedded in paraplast. Sections were collected at 10 microns.

Paraplast sections on slides were heated to 60–65°C on the slide warmer (30–60 min), rinsed with xylene and rehydrated through an ethanol series. For prehybridizations, slides were treated with proteinase K (0.5 μg/ml), post-fixed in 4% paraformaldehyde, acetylated in acetic anhydride and prehybridized for 1 hour at room temperature in hybridization buffer (50% Formamide, 5× SSC, 5× Denhardts reagent, Baker’s Yeast RNA, Herring sperm DNA). The riboprobes were diluted in hybridization buffer, denatured, and chilled on ice; 100 μl of diluted riboprobe was added per slide, the slides were covered with Parafilm M and incubated in a moist chamber at 60°C overnight. After hybridization, the Parafilm was removed in 5× SSC at 60°C, and the unhybridized riboprobe was degraded by incubating the slides at 37°C (30 min) in 20 μg/ml RNase A. The slides were washed with standard sodium citrate (SSC) series, with increasing stringency. The slides were blocked in 10% heat inactivated goat serum and incubated with anti-DIG-Alkaline Phosphatase-conjugated antibody (Roche) diluted 1:1000 in blocking buffer in a humidified chamber at 4°C overnight. The alkaline phosphatase activity was detected using nitro-blue tetrazolium chloride (NBT) and 5-bromo-4-chloro-3'-indolyphosphate p-toluidine (BCIP) (3–10 hrs). Stained tissues were imaged by Nikon (Melville, NY) fluorescent microscope and photographed using a digital camera (Photometrics CoolSNAP) system equipped with IPLab imaging software. Statistical analysis of images for staining was carried out by analySIS FIVE Digital Imaging Solutions Software (Soft Imaging System GmbH, Johann-Krane-Weg, Münster, Germany).

### Dual Immunofluorescence staining

The sections (10μm thick) were fixed in 4% paraformaldehyde in PBS (10 mM phosphate, pH 7.4) for 10 min after deparaffinization. Endogenous peroxidase was blocked in 3% H_2_O_2_/PBS for 15 min and permeabilized with PBS containing 0.1% Triton X100 for 30 min. The slides were rinsed three times in PBS-Tween 20 for 5 min each. After rinse, the sections were incubated with serum blocking solution for 1 hr. and subsequently incubated with the primary antibodies, ZEB2 (1:50, SIP1, polyclonal goat anti-human, SC-18392, Santa Cruz) and Clone E29 (1:100, monoclonal mouse anti-human epithelial membrane antigen, Dako) in blocking buffer for 3 hr at room temperature. The sections were washed three times in PBS-Tween 20 (0.1%) for 5 minutes. Alexa fluor 488-conjugated anti-goat IgG (1:1000, Invitrogen) and Cyanine 3 anti-mouse (1:1000, Jackson ImmunoResearch) were used as secondary antibody for 1hr at room temperature. Nuclei counterstaining was performed with 300 μl 4,6-diamidino-2-phenylindole (DAPI) per slide for 1 min. Coverslips were mounted with anti-fade fluorescent mounting medium. All images were visualized on LSM700 Confocal microscope with ZEN software.

### Northern blot

Five μg of total RNA in 2X RNA loading buffer (Thermo Scientific) was denatured for 5 min at 68°C and loaded on 1% formaldehyde agarose gel. RNA integrity was confirmed using Ethidium Bromide-stained gels. Total RNA was transferred to a Hybond-NX (GE Healthcare) and cross-linked to the membrane using UV. The membrane was prehybridized for 30 min at 37°C in hybridization buffer (0.25M Sodium Hydrogen phosphate, 1mM EDTA, 10% SDS, 0.5% blocking reagent) and hybridization was carried out overnight at 37°C. The membrane was then washed twice in 2X SSC/0.1% SDS at 65°C. The oligonucleotide probes (S1 Table) were labeled with DIG Oligonucleotide tailing kit, 2^nd^ Generation (Roche) and the signal intensities were analyzed using the ChemiDoC-IT Imaging System.

### RNA sequencing

To generate a single RNAseq library that comprises RNAs detected in both normal and tumor samples, total RNA (16.3 μg) from three breast cancer patients (Asterand plc Donor ID: 42937, 42954, 44739) that included both cancer and RNA matched normal tissue were pooled. The sequencing sample was prepared by removing ribosomal RNA using Ribo-Zero rRNA removal kit (Epicentre Biotechnologies). The library was prepared from rRNA-depleted RNA (550 ng) using TruSeq RNA Sample Preparation Kit (Illumina), omitting polyA selection to include non-polyadenylated RNA at Tufts University Core Facility, Genomics. Paired-end 50 base-pair (bp) sequencing was performed on Illumina HiSeq 2000 (TUCF, Genomics).

Since standard RNA-Seq libraries ignore directionality in transcription, a strand-specific RNA library was also prepared to complement the TruSeq library using the directional mRNA-Seq Sample Preparation protocol (Illumina Part # 15018460 Rev.A). To retain non-polyadenylated RNA, total RNA (1 μg) from matched normal and tumor samples from two patients (Asterand plc Donor ID: 45972, 46433) was used without polyA selection and fragmented using Fragmentation Reagents (AM8740, Ambion). The RNA clean-up using RNeasy MinElute column (QIAGEN), Phosphatase (M0289S, QIAGEN) treatment, T4 Polynucleotide Kinase (M0201S, QIAGEN) treatment, adapter ligation using components from the Small RNA v1.5 kit (Illumina), reverse transcription, PCR amplification, and cleanup using AMPure XP beads (Beckman Coulter) were performed as suggested by manufacturer. To deplete highly abundant DNA molecules from sources such as rRNA and tRNA, DSN Normalization protocol (Illumina Part # 15014673 Rev. C) was performed using the DSN Kit (EA001, Evrogen). The resulting library (14 μl of 45 ng/μl) was sequenced on Illumina Genome Analyzer II, 36 bp single-read run.

### Statistical Analysis

To compare the expression of the tested regions in normal and tumor samples, *p*-values were calculated using two-sample Welch t-statistics (unequal variances, two-tailed), adjusted for multiple testing using the multitest method (R package), using a total of 100,000 permutations. Mann–Whitney–Wilcoxon test was used to evaluate statistical significance for the differences observed for the coding-potential scores between intronic and exonic read clusters.

## Results

### GTA analysis reveal intronic regions associated with breast cancer

To identify novel genes that are important to breast cancer, total RNA samples (matched pairs from 4 patients) (S1 File) were purchased from Ambion and Asterand plc and used to generate disease versus control cDNA samples. These cDNAs were used to screen GTA arrays comprising ~80 million distinct microarray probes that target most of the human genome. These probes, distributed across 14 arrays consisted of both match and mismatch probes. Computational analysis of the resulting ~650 million probe intensities revealed a total of 338 differentially expressed gene regions consisting of 219 potentially novel gene regions (S2 File) and 119 known exonic regions. The potentially novel gene regions, ranged from a length of 220 nt to 5446 nt, with an average size of 1118 nt. Intriguingly, 97 of the 219 candidate gene regions derive from chromosome 2 (Chr2) and 75 regions are from Chr5. In comparison, Chr1, the largest chromosome, yielded only seven candidate gene regions associated with breast cancer.

**Fig 1:**
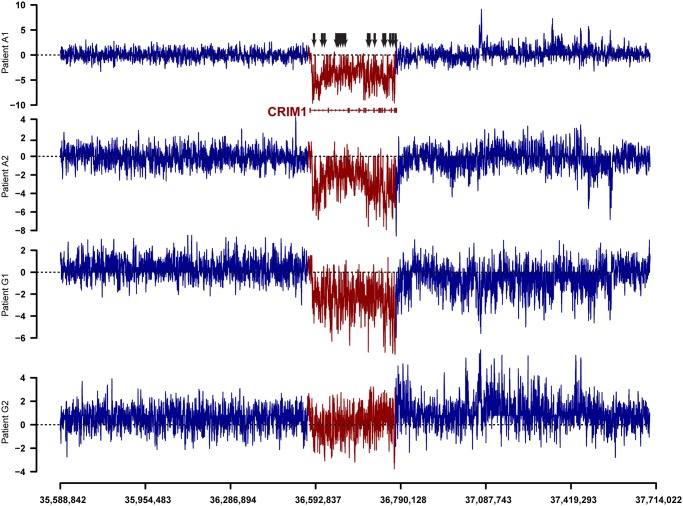
Tiling array difference signals between normal and tumor samples for a long candidate region relevant to breast cancer biology. The entire 195 kb long CRIM1 locus (red) trends toward downregulation in tumor samples. The marked CRIM1 intronic locations (arrows) that manifest reduced probe intensity signals in tumor were validated by PCR validations. Comparison of the CRIM1 locus to both its neighborhood, and its gene structure containing small exons (boxes) and long introns (lines) underscores the high degree of downregulation seen at multiple intronic locations. The tiling array signal intensities are relative intensities that are unique to each sample and hence should not be compared across the four different samples.

Despite performing a comprehensive genome-wide search for breast-cancer associated genes, the majority (188) of the 219 candidate regions correspond to introns of known genes. Many of these locations are clustered in close proximity to one another. For example, 19 tightly clustered regions down-regulated in the tumor samples are located within the introns of the CRIM1 gene (Fig. 1). High levels of clustering are found in introns of other genes, including SMA4 (16 clusters on Chr5), a complex 900 kb long locus annotated as a pseudogene, and ZEB2 (9 clusters on Chr2), a protein-coding gene that is down-regulated in a variety of cancers [16–18] and is a causal factor in the developmental disorder, Mowat-Wilson syndrome [19]. Differentially regulated genomic clusters also occur in regions that do not have a functionally annotated gene. For instance, Chr5 yielded an unannotated cluster of six up-regulated regions within 10 kb (Chr5:34198635–34208794), and recent studies have revealed multiple lncRNA [20] transcripts also from this region.

### Intronic regions have potential as useful breast cancer markers

**Fig 2:**
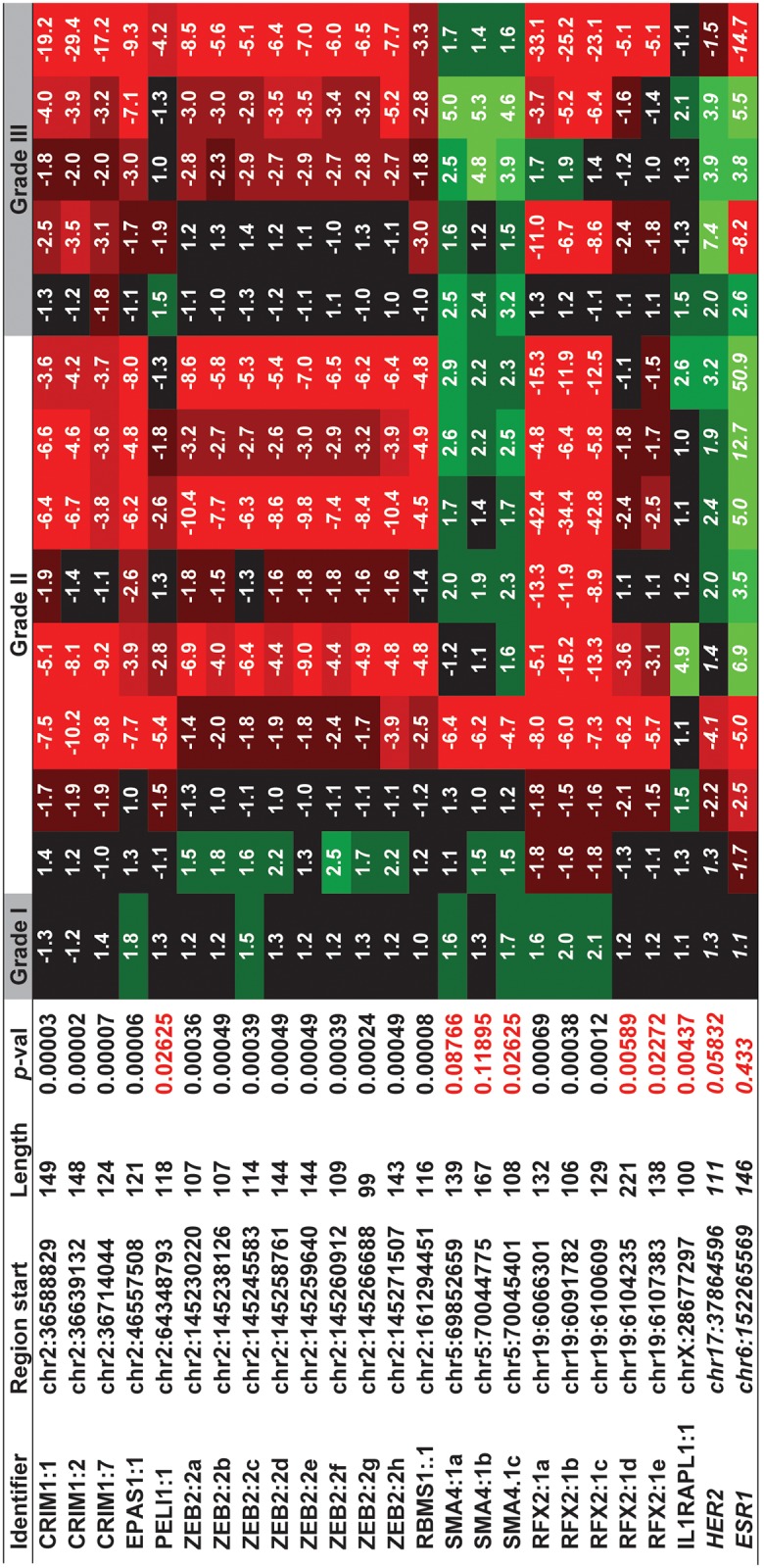
Gene expression patterns of candidate regions in matched-pair samples of breast cancer. Quantitative PCR validations of twenty eight independent patient samples (14 normal-tumor pairs) of progressive aggressiveness (grades I-III) were profiled. Of the locations that are differentially regulated in tumor (*p*<0.001); seven candidates (red) do not pass the moderately stringent *p*-value cutoff, some of these locations manifest reasonable statistical significance (*p*<0.01) and hence may be useful for further studies. The name of the host gene followed by the number of the intron that encompasses the tested region, followed by alphabetic numbering to differentiate the different regions tested within the intronic region are indicated (Identifier). For example, the ZEB2:2c region represents the third (c) candidate tested (spanning 114 bp, starting at chr2:145245583), which is located in the second intron of ZEB2, while CRIM1:7 represents the only candidate located within the seventh intron of CRIM1 (spanning 124 bp, downstream of chr2:36714044). Curiously most of the regions tested are located within the first intron of the host gene. *HER2* and *ESR1* which is generally overexpressed in more aggressive breast cancer, is used as an internal control.

The list of 219 candidates was further refined to a set of 123 regions by evaluation of the evolutionary conservation of the candidate regions in mammals, using the UCSC genome browser tracks, as well as their sequence similarity to mRNAs known to be involved in cancer. To validate the expression patterns, qRT-PCR was performed in an independent set of matched-pair tissues from four different patient samples by selecting representative unique candidate regions that are genomically clustered. This resulted in the identification of 23 regions that were either consistently up- or down-regulated in tumor. The expression patterns of these 23 regions were then validated in an independent set of 28 different patient and control samples (14 matched-pair tissues) (S2 File). The expression pattern for Human Epidermal Growth Factor Receptor 2 (HER2/neu, Fig. 2) and the estrogen receptor alpha (ESR1), known breast cancer markers, were used as controls. The 23 candidate biomarker regions fall within the introns of seven protein-coding genes (CRIM1, EPAS1, PELI1, ZEB2, RBMS1, RFX2, and ILRAPL1) and the intron of an uncharacterized RNA, SMA4. Although these genes do not have an established role in breast cancer, they are known to affect critical processes related to development or cell proliferation. For instance, ZEB2, a 1.2 kb mRNA derives from a very long transcriptional unit that spans over 136 kb and is thought to be a tumor suppressor [18, 21]. Intriguingly, eight of the 23 regions tested reside within well separated regions of ZEB2, loci that are in most cases over 4 kb apart.

RT-PCR analyses revealed that the many of the tested regions are strongly differentially regulated in the majority of the tissues (*p*<0.001). Candidates that exhibited the strongest association with breast tumors were regions from the longest introns of RFX2, EPAS1, RBMS1, ZEB2, and the three different introns of CRIM1. Proximal regions frequently manifest similar expression patterns. For example, the expression of all three CRIM1 regions are well correlated within both normal and tumor tissue types, yielding a correlation coefficient (r) of 0.95 between the first two regions (CRIM1:1 vs CRIM1:2) that are separated by over 50 kb and r = 0.77 for the first and third loci (CRIM1:1 vs CRIM1:7), separated by ~75 kb. The coordinated expression patterns that range from correlation coefficients close to 1.0 to more modest correlation numbers (e.g., 0.77 for CRIM1:1 vs CRIM1:7) imply that introns of the host genes harbor multiple exons, some of which are linked by a single transcript (e.g. CRIM1:1 vs CRIM1:2) while others likely represent distinct transcripts (e.g. CRIM1:1 vs CRIM1:7).

### Multiple intronic transcripts are aberrantly regulated in epithelial cells

Since most breast cancers originate from normal epithelial cells, we sought to investigate whether the intronic target regions were differentially regulated within epithelial cells. RNA in situ hybridization (ISH) was performed on paraffin-embedded matched-pair tissues isolated from breast cancer patients. Riboprobes were designed to detect unique non-repetitive regions within introns of CRIM1, RFX2, and ZEB2. To evaluate whether the expression patterns of these intronic regions are different from those of the host genes, we also designed probes against annotated exonic regions of these genes. Epithelial cell types in these experiments were identified by their characteristic cell morphology under 400X magnification of Nuclear Fast Red-stained nuclei. Consistent with the observed down-regulation of RFX2, ZEB2, and CRIM1 regions using qRT-PCR, in-situ hybridizations with probes corresponding to these genomic loci clearly reveal that these loci are down-regulated in tumor epithelium (Fig. 2 and S2 Fig.).

**Fig 3:**
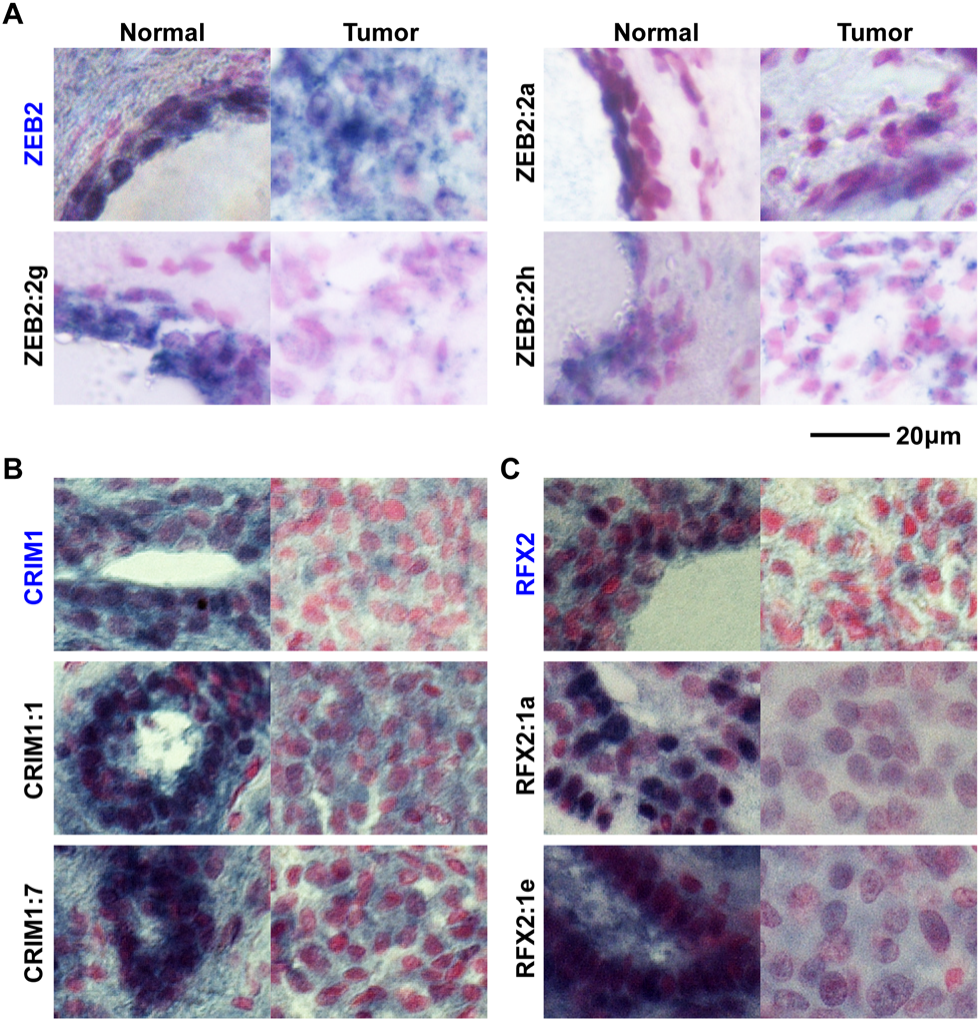
Examples of candidate intronic ncRNAs that are downregulated in breast cancer tissues, compared to the expression patterns of their host genes. In situ hybridization of the cognate host gene mRNAs (in blue) for ZEB2 (**A**), CRIM1 (**B**) and RFX2 (**C**), and the candidate intronic ncRNA (Black) in sample-matched normal (left panels) and cancer tissues (right panels). RNA transcripts are blue/black and tissue is counterstained with nuclear Fast Red. Among the seven intronic and three mRNA loci tested, a few intronic loci such as RFX2:1a, CRIM1:7, and ZEB2:2g intronic regions show strong down-regulation in tumor epithelial cells, compared to control tissue. The comparisons of normal and tumor expression for Zeb2 variants illustrates that in tumor tissues that lose epithelial characteristics, canonical Zeb2 mRNA is still present. On the other hand intronic regions, such as Zeb2:2g, show robust expression in normal control tissue yet are greatly reduced in tumor tissues. Therefore the candidate intronic RNAs are downregulated in tumor cells compared to their cognate control RNAs.

**Fig 4:**
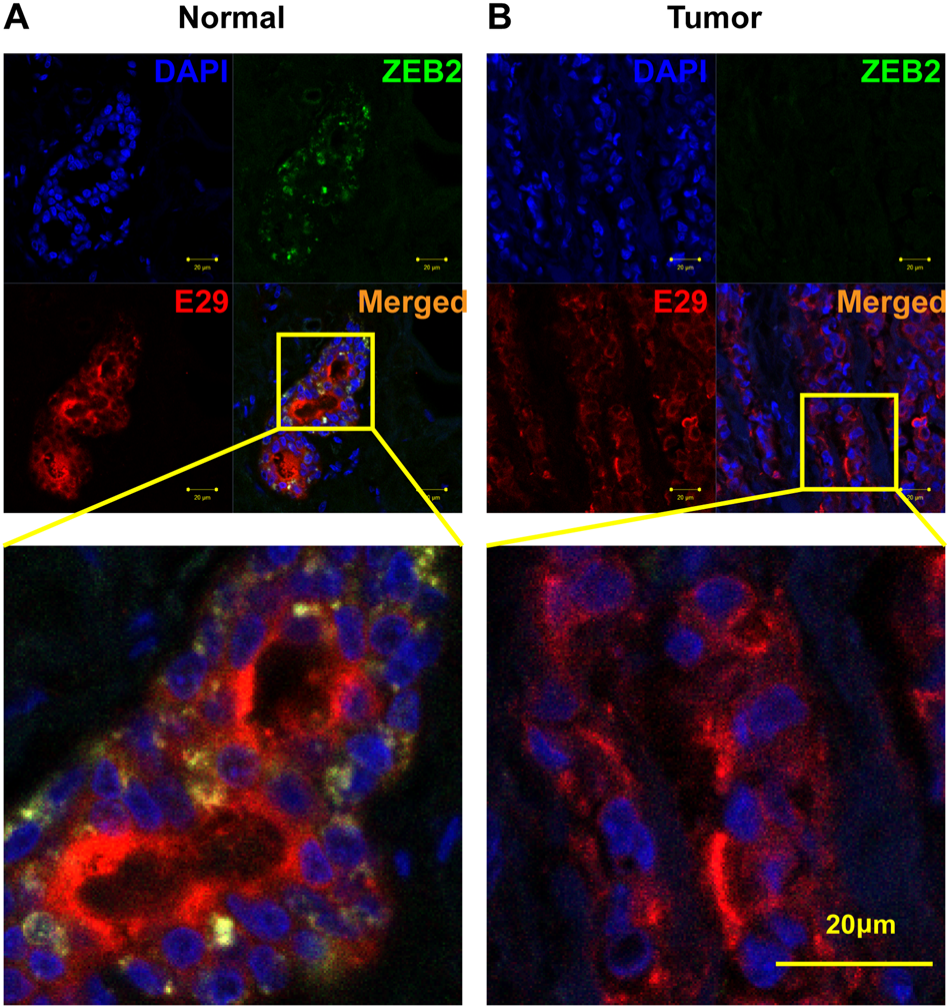
Downregulation of ZEB2 protein in epithelial cells. DAPI (blue) and E29 (red) staining are used to mark nucleus and epithelial cells of normal (**A**) and breast cancer (**B**) tissue, respectively, from a matched-pair invasive ductal carcinoma sample. Downregulation of ZEB2 (green) protein in breast epithelial cells (**B**) supports the idea that it is a target of aberrant gene regulation in breast cancer.

Since multiple differentially expressed regions within the 136 kb long ZEB2 were detected by both tiling arrays and qRT-PCR, and because ZEB is a tumor suppressor gene that regulates epithelial-mesenchymal transitions during development and tumorigenesis [18, 21, 22], the expression patterns of ZEB2 transcripts were analyzed in more detail. Similar to the disparate expression levels that we observed within the RFX2 locus (Fig. 3), another extraordinarily long (171 kb) transcript from various intronic regions within the long (~136 kb) ZEB2 gene frequently manifest dissimilar expression patterns. In normal tissue, both ZEB2 and its intronic probes (Fig. 3, ZEB2:2g, ZEB2:2h) are robustly expressed in breast epithelium. In breast cancer samples, these intronic transcripts and ZEB2 are down-regulated in epithelial cells. However, ZEB2:2g and ZEB2:2h transcripts are expressed at a lower level than the ZEB2 mRNA (Fig. 3). To confirm that the ZEB2 region was misregulated in breast cancer epithelial cells, we examined the expression pattern of the ZEB2 protein using immunohistochemistry. To identify epithelial cells, double immunofluorescence staining was performed on normal and breast cancer tissue sections with the epithelial cell marker MUC1 (E29) and the ZEB2 protein. Consistent with the qRT-PCR results, ZEB2 protein is visibly down-regulated in breast epithelial cells (Fig. 4). These results support the notion that the unusually long transcriptional unit of ZEB2 is frequently misregulated in cancer. Some of the ZEB2 intronic regions such as ZEB2:2g seem to serve as better markers of misregulation of the ZEB2 locus than the canonical ZEB2 exonic regions, a pattern we also observed for transcripts from the CRIM1 and RFX2 locus (Fig. 3).

**Fig 5:**
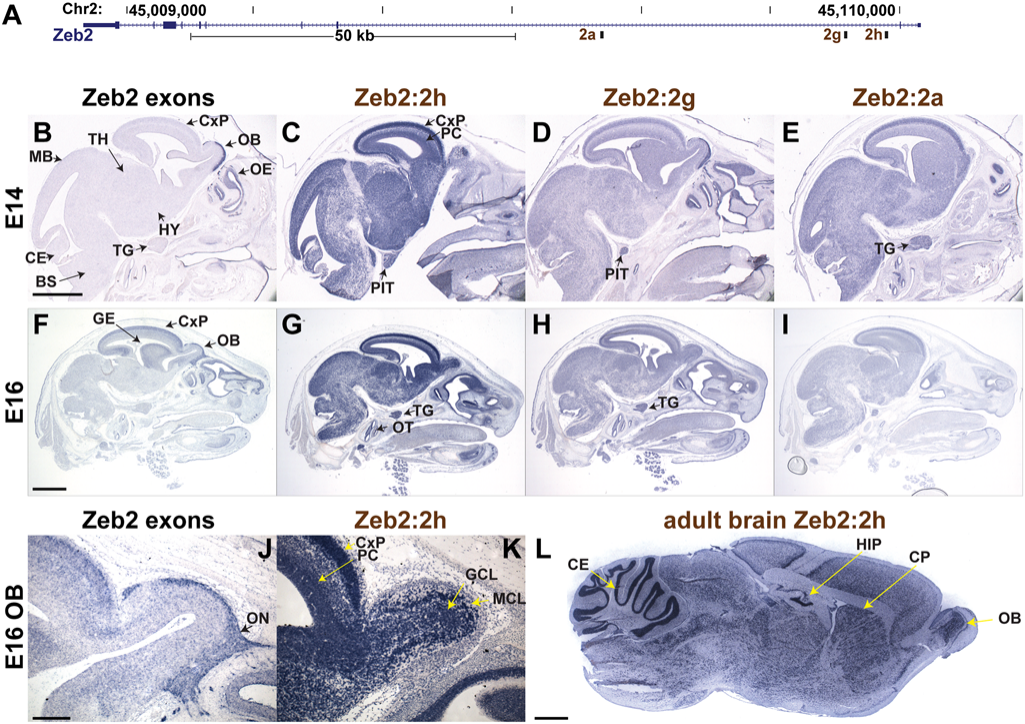
ZEB2 intronic sense transcripts manifest disparate expression patterns during development in mice. **A)** Intronic riboprobe to regions 2a, 2g, and 2h within ZEB2 gene were used to detect sense-strand RNAs in mouse embryo head at E14 (**B-E**). Two different exon regions were used as internal controls, one within 3’ UTR (**B**) and another corresponding to the cDNA riboprobe used in the Allen Mouse Brain Atlas at E16 (**F**). **B-I**) RNA *in situ* hybridization in sagittal head sections of E14 and E16 mouse embryos. Scale bar E14 = 0.5 mm; E16 = 1 mm. **J-K**) Sagittal images of the olfactory bulb of E16 embryos highlighting the complementary expression pattern of ZEB2 in the olfactory nerve (ON), and Zeb2:2h in the mitral (MCL) and granule (GCL) cell layers. Scale bar = 100 μm. **L.** Sagittal section, RNA *in situ* hybridization detecting region Zeb2:2h in the adult mouse brain. Scale bar = 1 mm. BS, brain stem; CE, cerebellum; CP, caudoputamen; CxP, cortical plate; GE, ganglionic eminence; HIP, hippocampus; HY, hypothalamus; MB, midbrain; OB, olfactory bulb, OE, olfactory epithelium; OT, otic sensory epithelium; PC, progenitor cells of the cerebral cortex; PIT, pituitary gland; TG, trigeminal gland, TH, thalamus.

The detection of transcripts embedded within intronic regions of ZEB2 raises the question whether these transcripts are simply byproducts of the primary ZEB2 mRNA or whether their transcription is independently controlled. We therefore compared their expression pattern to the endogenous ZEB2 gene. In addition, functionally important transcripts are usually conserved between mammalian species, so we first determined if these human transcripts are genomically conserved sequences in mice and whether they are also transcribed. Comparing the human genomic sequence of regions encompassing Zeb2:2a, Zeb2:2g, and Zeb2:2h (Fig. 5A) to the mouse sequence over an approximately 500bp stretch, reveal sequence identities of 69%, 89%, and 75%, respectively. To determine if these conserved regions are transcribed in mice and whether they manifest tissue-specific expression patterns, a common attribute of functionally important RNAs such as lincRNAs[23], we used ISH to examine their expression pattern in developing mice in middle stages of embryogenesis. Despite the high sequence identities shared by mouse and human sequences, the gene structure in mice as well as the function of these sequences could be very different, though an understanding of the general expression patterns of these sequences in mice could help inform future functional studies. To enable direct comparison of our results with emerging genome-wide ISH studies such as the mouse Allen Brain Atlas[24], mouse brain was used. ISH reveals expression of Zeb2:2a, Zeb2:2g, and Zeb2:2h in the brain (Fig. 5). During development, the canonical ZEB2 exonic regions are strongly expressed in the olfactory epithelium and the olfactory nerve (Fig. 5B and J), as well as enriched in the cortical plate and the ganglionic eminence (Fig. 5F). The hypothalamus, midbrain and hindbrain express lower levels of the ZEB2 transcript. In contrast, the intronic regions show broader expression patterns, suggesting that Zeb2 and its intronic transcripts are under different modes of regulation. Zeb2:2h is highly expressed in neurons throughout the brain with higher levels of expression in the cortical plate, ganglionic eminence, hypothalamus and olfactory bulb (Fig. 5C, G and K). In the cerebral cortex, expression of Zeb2:2h is higher in the mature neurons in the cortical plate than in neuronal progenitors (Fig. 5C and K). The difference in expression is especially striking in the developing olfactory bulb, where the ZEB2 is enriched in the olfactory nerve, while Zeb2:2h shows strong expression in the developing mitral and granule cell layers (Fig. 5J-K). Outside the central nervous system, Zeb2:2h is uniquely enriched in the otic sensory epithelium and in the trigeminal gland. Further, the expression of Zeb2:2h in the brain persists into adulthood, where it is enriched in the granular layer of the cerebellum, in the dentate gyrus of the hippocampus, in the olfactory bulb, and in the caudate putamen (Fig. 5L). Cell-type localization patterns of Zeb2:2g and Zeb2:2a, share similarity with both the canonical ZEB2 and Zeb2:2h (Fig. 5D-E and H-I). They are similar to Zeb2:2h in the cortex, hypothalamus, trigeminal, and otic sensory epithelium. In the olfactory bulb they are enriched in the olfactory nerve, similar to the endogenous ZEB2. These observations are consistent with the idea that various sub-regions within the long ZEB2 loci are disparately regulated.

### Intronic transcripts are likely transcribed in the sense strand, are mostly non-polyadenylated, and are ncRNAs

**Fig 6:**
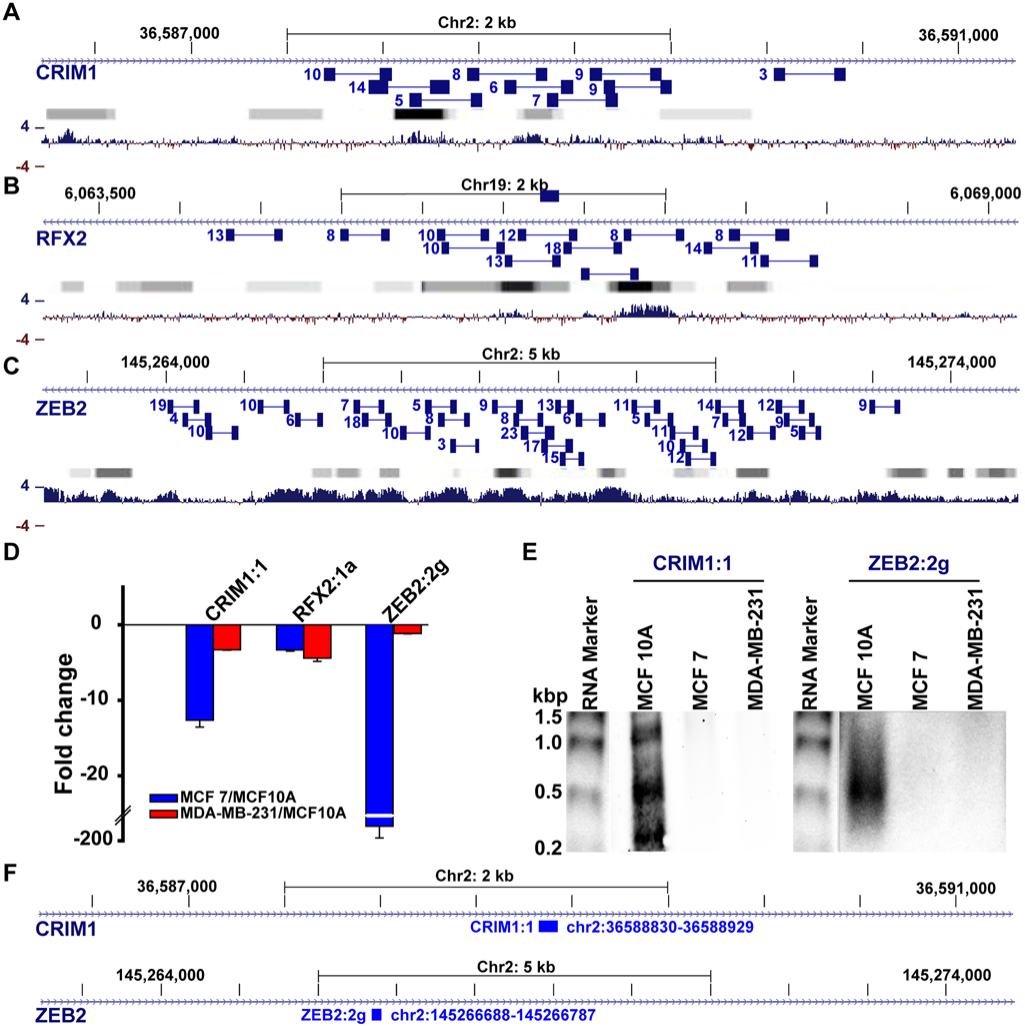
Paired-end sequencing read patterns confirm the presence of intronic transcripts. **A-C**) The number of reads obtained from overlapping paired sequence reads (connected blue boxes) are indicated next to each paired read. Gradient bars (grey to black) represent known transcription factor binding sites obtained from ENCODE ChIP-Seq experiments, generated using UCSC genome browser; darker boxes represent increased transcription factor binding signal strengths across ENCODE experiments. Bottom bar plotgraphs (blue) represent the extent (4 = high, -4 = low) of evolutionary conservation in placental mammals. Reads obtained at locations of CRIM1:1 (**A**), RFX2:1a (**B**), and ZEB2:2g (**C**) reveal clusters of transcript regions that are generally located next to known transcription factor binding sites (Gradient bars) and highly conserved locations (blue). **D**) qPCR detection of differential expression of CRIM1, RFX2, and ZEB2 intronic transcripts in model breast cancer lines (MCF-7 and MDA-MB-231), in comparison to the model epithelial (normal) cell line, MCF10A. **E**) Analysis of CRM1:1 and ZEB2:2g locations by northern blot reveals the overabundance of novel transcripts that range from ~300–1000 nts for CRIM1:1 and ~500nts for MCF10A. **F**) the location of probes used for the northern blot.

Regions detected using oligonucleotide hybridization based methods such as tiling arrays, PCR, and ISH may correspond to alternative genomic regions that simply share similarity with the inferred genomic regions or represent full-length introns that are unspliced in the pre-mRNA. Although the latter possibility is unlikely because pre-mRNAs as well as spliced out introns are transient RNA species that are rapidly degraded[25], it is possible that certain introns are stabilized by protein complexes to levels comparable to short-lived mRNAs[26]. To test whether these genomic regions are intron-derived RNA transcripts, we performed paired-end deep sequencing using Illumina HiSeq and used the results to infer the transcriptional units of the sequences. Many paired-end reads were sequenced from intronic-regions (CRIM1, ZEB2, RFX2) that overlap the intronic regions validated by PCR and ISH (Fig. 6A-C). The intronic reads frequently form discrete clusters, suggesting that these clusters might represent multiple intronic RNAs. To confirm these observations, we used northern blot to test for the presence of intronic RNAs that are much smaller than the expected size of the unspliced introns. Consistent with sequencing and PCR results, northern blots reveal abundant short transcripts derived from both CRIM1:1 and ZEB2:2g regions, processed in MCF10A, a normal human mammary epithelial cell line, but are downregulated in both MCF7 and MDA-MB-231, two aggressive breast cancer cell lines (Fig. 6D-F). To determine whether the sequences derive from the sense or anti-sense strand of the introns, we performed strand-specific deep sequencing. In the neighborhood (± 50 nt) of the 23 regions verified by PCR (Fig. 2), considerably more (89 *vs* 6) strand-specific reads were obtained from sense strands than anti-sense strands, substantiating that the intronic reads correspond to sense transcripts.

The confirmation of novel intron-derived RNAs by northern blot and supported by paired-end sequencing clusters raised the question as to whether these RNAs are polyadenylated like mRNAs and whether they code for proteins. Direct RNA Sequencing (DRS) is a sequencing based approach that can detect polyadenylation sites in, and is able to provide an overall view of the polyadenylation patterns of many genes simultaneously [27]. We performed Direct RNA Sequencing of MCF7 cells and two samples of breast cancer [27]. The polyadenylation sites identified by DRS did not map to any of the transcriptional units inferred by sequencing or by northern blot, suggesting that the breast-cancer associated regions we identified are non-polyadenylated transcripts. While DRS might not capture the polyadenylation state of every transcriptional unit due to the depth of the sequencing reads, the observation that none of the well-expressed transcriptional units yielded a DRS signal suggests that the majority of these regions do not represent polyadenylated transcripts. To establish whether these clustered regions correspond to alternative transcript variants of the host gene or other protein-coding genes, we analyzed all read paired-end read clusters for their coding potential and found that the majority of the intronic transcripts are predicted to represent ncRNAs (S1 Fig). While the gene prediction algorithms are not fully accurate, the striking differences in the coding potential scores of these regions compared to other exonic regions suggest that most of these are ncRNAs. Taken together, these observations indicate that the intronic regions associated with breast cancer represent non-polyadenylated ncRNAs that are transcribed in the same direction as their host genes.

## Discussion

A few biomarkers such as BRCA1/2 and HER-2/neu have been very useful in guiding therapeutic interventions in breast cancer [12]. Genetic markers that can help in the diagnosis, prognosis, or treatment of breast cancer are highly desired to improve both breast cancer treatment and understanding of the underlying biology. With the emergence of high-throughput gene expression quantification platforms such as deep-sequencing, it has become feasible to find novel genes to serve as markers or targets in cancer treatment. We performed a comprehensive search to identify novel genomic associations in the breast cancer transcriptome. Surprisingly, despite the fact that as much as 90% of the genome is transcribed and the majority of full-length transcriptional units remain undefined in intergenic regions, intronic locations rather than intergenic regions emerged as key candidates associating with breast cancer. It is possible that such enrichments may be because intronic regions are more transcriptionally active. Nevertheless, it is useful to note that while only a minority (15%) of genes are more than 100 kb of genomic sequence, the identified locations are biased towards unusually long genes that span over 100 kb [28]. An original list of ~220 candidate regions from GTA analysis was narrowed down to 16 breast-cancer associated regions that are located within the introns of five well-characterized genes. Among these, the down-regulation of at least three regions (CRIM1:1, RFX2:1a, and ZEB2:2g) in breast epithelial cells suggest that they may be useful as clinical markers for pathologic evaluation of cancerous breast cells from tissue biopsies.

Among the CRIM1:1, RFX2:1a, and ZEB2:2g regions that derive from well characterized gene loci, the ZEB2 locus is particularly interesting. The canonical ZEB2 gene, recently recognized as a tumor suppressor [18, 21], is known to regulate the Epithelial–Mesenchymal Transition (EMT) program, a pathway that changes adherent epithelial cells into less adherent migratory cells, a process critical for oncogenic progression and metastasis of tumor cells [22]. Consistent with ZEB2 as a tumor suppressor, several reports have documented down-regulation of ZEB2 mRNA in multiple cancers including those of brain, breast, colon, liver, skin, and pancreas [16–18, 29, 30]. In cancers of liver and pancreas, ZEB2 is epigenetically silenced [16, 17]. However, the observations that ZEB2 introns are transcribed in modular patterns, potentially generating many ncRNAs, and that such intronic transcripts are strongly associated with breast cancer add a new direction to the analysis of the ZEB2 locus. These findings are very intriguing when considering the recent discovery that ZEB2 mRNA acts as a competitive endogenous RNA (ceRNA) to repress the PTEN tumor suppressor gene by sequestering miRNAs that would otherwise down-regulate PTEN [18]. Due to extensive transcription detected in this locus, some of the intronic ZEB2 RNAs could also act as ceRNAs that sequester regulatory factors, such as miRNAs or other proteins. In addition to being a tumor suppressor, ZEB2 is critical for proper brain development. ZEB2 mutations cause the Mowat-Wilson syndrome, a disease characterized by mental retardation, Hirschsprung disease, microcephaly, and distinct facial malformations [19, 31–34]. Consistent with the role of ZEB2 locus in brain development, we find ZEB2 intronic transcripts in mouse brain with unique expression patterns for different intronic regions that seem to express ncRNAs. We note that the expression patterns of ZEB2 regions in mice may not accurately represent the biology of ZEB2 regions in human. However, our analysis using both human and mouse tissues seems to point to an unusual level of transcriptional activity and regulation at ZEB2 intronic regions. Our results reveal that the ZEB2 locus, implicated in multiple diseases including cancer, is a far more complex region than is currently understood.

The detection of intronic transcripts that have disparate expression patterns is intriguing, particularly in the context of cancer. To gain insights into the cause of cancer-associated transcripts that derive from long introns, we need to evaluate the following three possibilities. First, these long introns may be stabilized during transcription as a single pre-mRNA or as different pre-mRNAs harboring various unspliced introns. However this would imply that different unspliced long pre-mRNA isoforms accumulate in normal cells while they are down-regulated in cancer cells. This seems unlikely not only because such long pre-mRNAs would be unstable, but because it seems more logical to expect the contrary, up-regulation of aberrantly spliced transcript isoforms in cancer [35]. Furthermore, paired end sequencing results clearly reveal many intronic locations that are more expressed than the canonical exons. Such intronic locations also appear as overlapping and tightly clustered regions that indicate the presence of transcript units independent of introns, consistent with our northern blot results and other studies[36]. A second possibility is that the spliced introns may be processed as RNA fragments of varying stability, which would lead to the accumulation of different intronic transcripts in the cell. Third, long intragenic RNAs such as lncRNAs may be transcribed as nested-genes at these locations. The latter two possibilities are consistent with both the observed down-regulation of intronic transcripts in tumors, the different expression patterns of various intronic regions within a given gene locus, and the clustered pattern of abundant paired-end reads. Indeed, processing of introns to generate more stable RNAs is a well-known phenomenon in the miRNA and the related mirtron pathway where miRNA-embedded introns are processed by Drosha in either a co-transcriptional manner [37] or after lariat de-branching [38, 39]. More studies are needed to differentiate between the two possibilities and determine the mechanisms behind the misregulation of the newly identified intronic regions in cancer. These novel breast-cancer associated intronic regions provide a rich set of novel targets for the diagnosis or treatment of breast cancer.

## Supporting Information

S1 Fig Intronic reads were predicted to have little coding potential. Reads mapping to introns and exons in the 219 regions detected by tiling arrays were used for this analysis using the support-vector machine-based Coding Potential Calculator (cpc.cbi.pku.edu.cn). The two distributions are visibly different, with reads mapping to coding regions yielding significantly higher scores with means much higher than that of the intronic reads (inset; *p*<10^-16^).
(TIF)

S2 Fig Negative controls of ISH for Fig. 3. Negative control experiments that did not use RNA probes, indicate that background staining is minimal.
(TIF)

S3 Fig Statistical analysis of ISH for Fig. 3. Quantitative imaging and statistical analysis of in situ hybridization of breast tissues by Analysis FIVE Digital Imaging Solution Software. A comparative ISH of the cognate host gene mRNAs (in blue) for ZEB2 (**A**), CRIM1 (**B**) and RFX2 (**C**), along with candidate intronic ncRNA ISH (Black) in sample-matched normal (blue bar) and cancer tissues (red bar). Data represents mean ± S.D. Statistical significance was determined by One-way ANOVA test, where *p* values are *p<0.05 and **p<0.01.
(TIF)

S1 Table List of PCR primers, and probe sequences used for ISH and northern blots. (DOCX)

S1 File The characteristics for all human samples used in this study. (XLSX)

S2 File List of potentially novel gene regions associated with breast cancer. (XLSX)
