# Supplementary figures and images for: Genome-Wide Transcript Profiling Reveals Novel Breast Cancer-Associated Intronic Sense RNAs
Source: PLoS One. 2015 Mar 23;10(3):e0120296. doi: 10.1371/journal.pone.0120296 (PMC4370647; doi:10.1371/journal.pone.0120296)

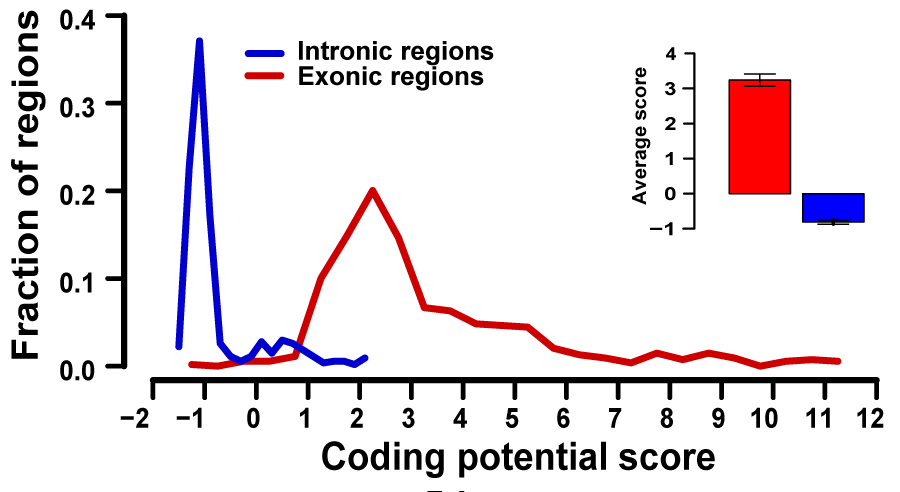

Supplement: S1 Fig — Reads mapping to introns and exons in the 219 regions detected by tiling arrays were used for this analysis using the support-vector machine-based Coding Potential Calculator (cpc.cbi.pku.edu.cn). The two distributions are visibly different, with reads mapping to coding regions yielding significantly higher scores with means much higher than that of the intronic reads (inset; p<10-16). (TIF) [file pone.0120296.s001.tif]

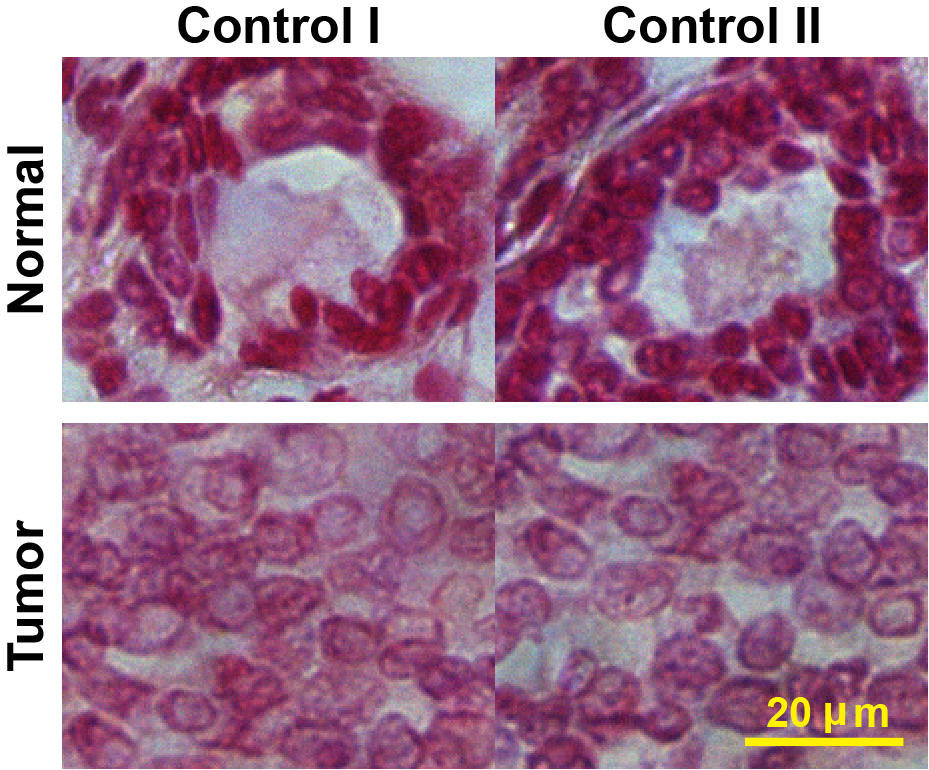

Supplement: S2 Fig — Negative control experiments that did not use RNA probes, indicate that background staining is minimal. (TIF) [file pone.0120296.s002.tif]

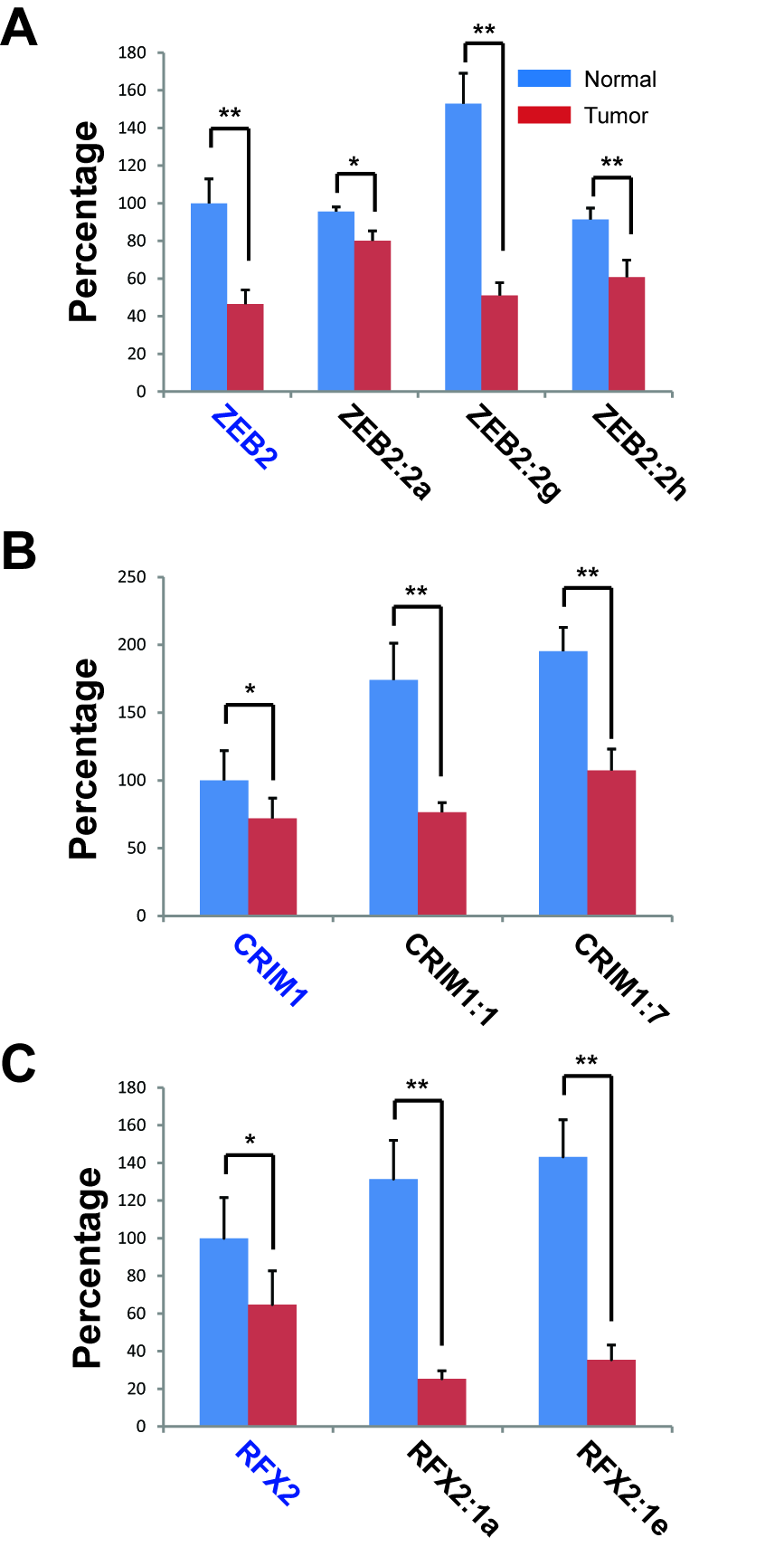

Supplement: S3 Fig — Quantitative imaging and statistical analysis of in situ hybridization of breast tissues by Analysis FIVE Digital Imaging Solution Software. A comparative ISH of the cognate host gene mRNAs (in blue) for ZEB2 (A), CRIM1 (B) and RFX2 (C), along with candidate intronic ncRNA ISH (Black) in sample-matched normal (blue bar) and cancer tissues (red bar). Data represents mean ± S.D. Statistical significance was determined by One-way ANOVA test, where p values are *p<0.05 and **p<0.01. (TIF) [file pone.0120296.s003.tif]
