# Supplementary material for: Genome-Wide Transcript Profiling Reveals Novel Breast Cancer-Associated Intronic Sense RNAs
Source: PLoS One. 2015 Mar 23;10(3):e0120296. doi: 10.1371/journal.pone.0120296 (PMC4370647; doi:10.1371/journal.pone.0120296)
Supplement: S1 Table — (DOCX) [file pone.0120296.s004.docx]

Table S1: List of PCR primers, and probe sequences used for ISH and northern blots.

| **Region** | **Forward Primer** | **Reverse Primer** |
| --- | --- | --- |
| **RT-PCR** |  |  |
| ACTB | TGCCCTGGCACCCAGCACAATGAAGAT | ACATCTGCTGGAAGGTGGACAGCGA |
| ESR1 | AAGAAGAACAGCCTGGCCTTGTCCCTGAC | TGCCAGGTTGGTCAGTAAGCCCATCATCG |
| ESR2 | AAAGCAAAGAGGGCTCCCAGAACCCACAG | GCAGAAAGATGAAGCCCAGGCTCCTGACA |
| GAPDH | ATGCCTCCTGCACCACCAACTGCTT | TGGCAGTGATGGCATGGACTGTGGT |
| HER2 | ACGTGCTCATCGCTCACAACCAAGTGAGG | CCATTGTCTAGCACGGCCAGGGCATAGTT |
| RPLP0 | TTGGGCTGGTCATCCAGCAGGTGTT | ACACTGGCAACATTGCGGACACCCT |
| CRIM1:1 | TCCCAGGCTATGGTGCAAGCAGAGATGATA | CCTGATACCAAACGTGCCATACGGAACACA |
| CRIM1:2 | TGCTTCCCGCACACTAGTGAAGCTGAC | ACCCGTAGCAGCTCTGGTCTACCTAAAGGG |
| CRIM1:7 | TGGATGAGACAACTCTTAGGCAACC | CAATGGTTGCAGCATAAAGAAGATGG |
| EPAS1:1 | AGTGCACTGTCCCTGAACTGAGGCT | AAGGCCACTGGGAACCCTGCTCTTT |
| PELI1:1 | ACTTTGGAAGGCCAAGGCAGGTGGAT | ATGTGCCACCACGCCTGGCTAATTT |
| ZEB2:2a | AACCTGGGCGTGCTCACAAAGAGGA | TCCTGGTACACACCACTCAAGGGCA |
| ZEB2:2b | ATGTTGCTTGGCCCTACTCTGTTTGC | TGGCAGACAGCATTCCTATCAGGGCAGAAA |
| ZEB2:2c | TGTCTCCATGAACGGGTGGGAGATCACAT | ACACAGATATATGAAGCTGGGTGGGCCTCT |
| ZEB2:2d | AGCCTAATGTCACACCCACTCCCAATCT | AATGGCCTAGCCCAGGACTCATTCAAGT |
| ZEB2:2e | GCACGCTTAGGGCAGGTAGAAATGTGG | AGGTGAGGAAGGTGGCAAAGGAGAG |
| ZEB2:2f | TGCACATGGATGATGCCAGCAAGGC | TGCTGATGCCCATTCGACAGGCACT |
| ZEB2:2g | ATTCTGAGTGTCTTTGGAGAGCCAGG | TTAACCTACCCAGCAATGCGGGAACA |
| ZEB2:2h | ACATTCTTGTCTGCGGGCAACTCGCTTT | TTAACCCATAGCGGGCAGTCAGTCTCCTT |
| RBMS1:1 | GGCAGGAGAATCACCTTGTTCATCCACT | GTAGGAACAGGAGGACCCTAAGGCATTT |
| SMA4:1a | GTGCTCACCGACAATGAAACATCTCCTC | GTCACTCCTTCTCCTTCAAGTACTGACCA |
| SMA4:1b | CCTTCCCTAGAGCAAGTATTTCAATGCAGTC | TCACCCAGTTCCAGACAAACCCAAGTCT |
| SMA4:1c | CAGAACTCAAGCTCCATGAGGAGA | ACCTGGAAAGGTTCTAGACACAGGG |
| RFX2:1a | AACCAGAAGGTGCTGGGTCCAATTTCCA | AGCACGATGCCTTCATTTCTGCTGCCTA |
| RFX2:1b | TGTTTCAGGAACTCTGAGTCTAAGCCGC | AAATGCCTACCTCCCGCAGTTCATTCCT |
| RFX2:1c | AAGTAGGCTCATGCAGGCCCACGTAAAT | AATTTCACGAACTGCCGGATGGTGGAGTCA |
| RFX2:1d | AATCCCAGCACTTTGGGAGGCTGAGGT | AGGCATCCACCACCACAACTGGCTA |
| RFX2:1e | TTTGGGAGGCCAAGGCGGTTGGATCATTT | TGAATAGCTGGGATTACAGGCATGCACCC |
| IL1RAPL1:1 | TCAGTTGCCTCCTGCAAAGCACCGATA | AAACTTCCCGTCCAGCGTTTGGTGCAA |
| **ISH** |  |  |
| CRIM1 | TTTCTATACAGTCACAACTGCA | GCCTGTTTATCTGCCTTTGTTC |
| CRIM1:1 | TCCCAGGCTATGGTGCAAGCAGA | TCCATGCAAAGGCAAGTCAGATCTT |
| CRIM1:7 | TGTTGAAGTGGCGTGCAAGGGG | GGCCTTTTCTGAAGGGCATGTCTCT |
| RFX2 | TGCGGATCTGCCGGGACACA | TACCCCTCCCTCCGCAGATGAC |
| RFX2:1a | ACATGTGAGCCAGCCAGGGC | CGGGAGTGGACTGCGTTGCC |
| RFX2:1e | CCAGCACCTGCATTTCTGGTGGA | TCTGCGTGAAGGGCACGCATT |
| ZEB2 | CATGCGAACTGCCATCTG | TATGCCTCTCGAGCTGGG |
| ZEB2:2a | AGCGGGGTGTGATTGGGGAGTC | TTCCTGGTACACACCACTCAAGGGC |
| ZEB2:2g | TTGGAGGGCTTATGAGCA | GGACACTTGCGGGGAA |
| ZEB2:2h | GACTGACTGCCCGCTA | CCCTTTGGGAATTGCTA |
| **RT-PCR** |  |  |
| CRIM1:1 | CCCTTCCACTCATTTTACCACCTCC | CCCAGGCTATGGTGCAAGCAGA |
| RFX2:1a | CCAGAAGGTGCTGGGTCCAA | AAGAGTTGAGCCCTGGCTGGC |
| ZEB2:2g | TCTGAGTGTCTTTGGAGAGCCAGG | CCTACCCAGCAATGCGGGAACA |
| **Northern blots** |  |  |
| CRIM1:1 | CACTAAATGTATTTCCCTTCCACTCATTTTACCACCTCCTACCCTCTTGTATTTACAGGCTAAAGCAACACTATCATCTCTGCTTGCACCATAGCCTGGG | |
| ZEB2:2g | ATTCTGAGTGTCTTTGGAGAGCCAGGATTTTATCTGCTGAGCGCAAGGGGCCAGGCACTCAAAGAGTTAAAGAGTGTTCCCGCATTGCTGGGTAGGTTAA | |
